# A computational account of joint SSRI and anti-inflammatory treatment

**DOI:** 10.3389/fimmu.2025.1472732

**Published:** 2025-04-25

**Authors:** Melissa Reneaux, Helen Mayberg, Karl Friston, Dimitris A. Pinotsis

**Affiliations:** ^1^ Centre for Mathematical Neuroscience and Psychology and Department of Psychology, City St. George’s —University of London, London, United Kingdom; ^2^ Psychology and Behavior Program, School of Liberal Studies and Media, UPES, Dehradun, India; ^3^ Department of Neurology and Neurosurgery, Icahn School of Medicine at Mt. Sinai, New York, NY, United States; ^4^ Wellcome Centre for Human Neuroimaging, University College London (UCL), London, United Kingdom; ^5^ The Picower Institute for Learning & Memory and Department of Brain and Cognitive Sciences, Massachusetts Institute of Technology, Cambridge, MA, United States

**Keywords:** immune system, inflammation, prefrontal cortex, subcallosal cingulate cortex, meanfield model, selective serotonin reuptake inhibitors (SSRIs), brain activity

## Abstract

**Introduction:**

Depression is a chronic disorder that impacts millions worldwide. Traditional treatments may not always work. Inflammation seems to be an underlying cause for chronicity and treatment non-response.

**Methods:**

We present a computational model that elucidates the interplay between inflammation, serotonin levels, and brain activity.

**Results:**

The model delineates how inflammation impacts extracellular serotonin, while cerebral activity reciprocally influences serotonin concentration. Understanding the reciprocal interplay between the immune system and brain dynamics is important, as unabated inflammation can lead to relapsing depression. The model predicts dynamics within the prefrontal cortex (PFC) and subcallosal cingulate cortex (SCC), mirroring patterns observed in depressive conditions. It also accommodates pharmaceutical interventions that encompass anti-inflammatory and antidepressant agents, concurrently evaluating their efficacy with regard to the severity of depressive symptoms Our model shows that for mild and moderate levels of depression anti-depressant agents or anti-inflammatory agents acting in isolation can bring serotonergic levels and brain activity to control levels. However, for severe depression only joint treatment of anti-depressant and anti-inflammatory agents can bring the serotonergic levels and activity to control levels.

**Discussion:**

This study is a first step to mechanistically understand the intricate link between the immune system and depression, the role of inflammation and potential treatments. It explores the impact of anti-depressant and anti-inflammatory drug treatments and assesses their relevance with regard to depression severity.

## 1 Introduction

Depression affects 4.4% of the world’s population (1, 2). Putative causes are multifactorial. They include monoamine depletion, anxiety, stress and inflammation, all of which mutually interact. These factors affect activity in distinct brain circuits regulating emotional behavior, including the dorsal raphe nucleus (DRN) and other infralimbic and prelimbic cortices and frontal areas (3). Depression is thus considered a circuit disorder (4–6). Studies have revealed the central role of circuits like the subcallosal cingulate cortex (SCC) (7–9), default mode network (5) and executive control network (10).

Depression is a chronic disorder. A significant number of patients experience depression relapse (11). Recurrent depression and disease progression are common (12). Treatments work often but not always (13). Some patients develop treatment non-response or resistance (14). One cause underlying disease chronicity and non-response is inflammation (15–17). Inflammation has profound effects on interoception and ensuing affective regulation (18, 19). It constitutes a threat to allostasis, i.e., the ability of the brain to resolve and pre-empt environmental and internal challenges to the body by adapting physiological parameters (20, 21). About a quarter of depressed patients exhibit inflammation (22). Some patients show resistance to anti-depressants but respond to anti-inflammatory drugs (23–25). In brief, depression is a circuit illness affected severely by inflammation. To understand its pathology and the link between inflammation and depression we developed a computational model. Previous work with similar models has described alterations in excitation to inhibition balance (26), the role of serotonin and other monoamines in depression (27, 28) and changes in the Hypothalamic Pituitary Adrenal (HPA) axis (29). From a purely theoretical standpoint (30), considers the complementary alterations of immunological sensitivity as an analogue of sensory attenuation.

Here, we focused on interactions between the immune system, the serotonergic system and brain activity. Cytokines are a key part of the immune system: they are chemicals used by immune cells for communication. They are elevated in inflammation and have altered levels in inflammation and depression (31, 32). Several cytokines including Interleukin 6 (IL6), C-reactive protein (CRP) and Tumor Necrosis Factor alpha (TNFα) have elevated levels in depression (15). TNFα is the most relevant for depression as it regulates extracellular serotonin levels (33–38). Peripheral administration of TNFα antagonist has been shown to improve depressive mood (39), reduce fatigue (40) and alleviate depression (41). Furthermore, TNFα receptor knock out mice show reduced anxiety-like behavior during immune activation (42).

Specifically, we modelled how TNFα affects neural activity in the prefrontal cortex (PFC) and the subcallosal cingulate gyrus (SCC). We focused on these areas because they show highly consistent depression-related abnormalities and are the main targets for treatment (43). Our model includes excitatory and inhibitory neuronal populations driven by NMDA, GABA and serotonergic currents, which evince the dynamics of 5HT1A and 5HT2A receptors. The amplitude of serotonergic currents was determined by extracellular serotonin concentration. In turn, this was modulated by changes in serotonin synthesis and reuptake due to inflammation. We quantified the impact of inflammation using the ratio of TNFα concentration in patients vs. control — that we called degree of inflammation (36). Finally, we introduced expressions that link this degree to serotonin synthesis and reuptake.

Technically, our model is a neural mass model based upon a set of stochastic differential equations describing the kinetics of serotonin and synaptic dynamics of coupled neuronal populations. Neuronal activity corresponds to the mean synaptic activity (modelled by synaptic gating variables, equipped with random fluctuations) at steady-state. Numerically, neuronal activity is quantified by the mean (and standard error of the mean: sem) over multiple solutions of the differential equations, for any given set of their parameters. The ensuing neural mass model predicts the impact of peripheral inflammation on depression for different cytokine and serotonin concentrations. It also explains how a joint Selective Serotonin Reuptake Inhibitors (SSRIs) and anti-inflammatory treatment might ameliorate depression.

## 2 Methods

We studied the impact of peripheral inflammation on a cingulo-frontal network associated with depression. We used a Dynamic Mean Field model (27). This includes two subnetworks for the prefrontal cortex (PFC) and the subcallosal cingulate cortex (SCC). The activity of both these brain regions is impacted in depression. Inflammation was modelled here through elevated levels of the cytokine TNFα.

The population firing rates of the PFC and SCC subnetworks are given by Equations 1, 2:


(1) $$r_{E}^{n}=\;H_{E}\left(I_{E}^{n}\right)=\;\frac{{ɡ}_{E}\left(I_{E}^{n}-\;I_{E}^{thr}\right)}{1-e^{\left(-d_{E}{ɡ}_{E}\left(I_{E}^{n}\;-\;I_{E}^{thr}\right)\right)}}\;\;\;$$

(2) $$r_{I}^{n}=\;H_{I}\left(I_{I}^{n}\right)=\;\frac{{ɡ}_{I}\left(I_{I}^{n}-\;I_{I}^{thr}\right)}{1-e^{\left(-d_{I}{ɡ}_{I}\left(I_{I}^{n}\;-\;I_{I}^{thr}\right)\right)}}\;\;\;\;$$

**Figure 1:**
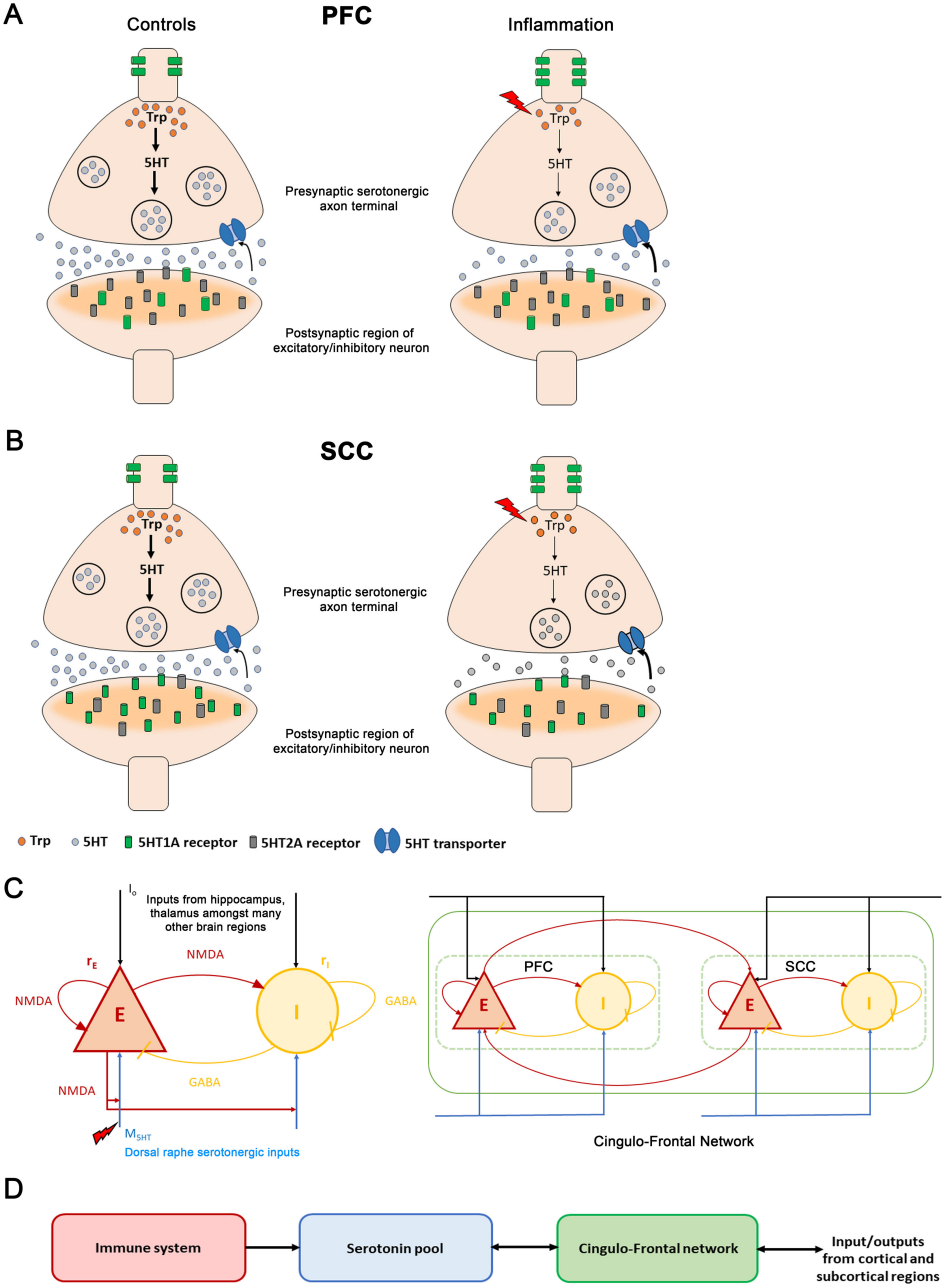
The immune-brain interaction**(A)** Changes between control and inflammation at a synaptic terminal in the prefrontal cortex (PFC). In control subjects (left panel), tryptophan (orange discs), a precursor of serotonin, is converted to serotonin (grey discs) in the presynaptic serotonergic neuron. Serotonin is then packaged in synaptic vesicles (large black circles). Synaptic vesicles move towards the synaptic bouton and fuse to the membrane releasing serotonin into the extracellular medium. The extracellular serotonin binds to the postsynaptic receptors (grey and green cylindrical structures) and signals a cascade of events in the postsynaptic excitatory/inhibitory neuronal terminal. The excitatory serotonergic 5HT2A receptors (grey) are abundantly present in PFC compared to inhibitory 5HT1A receptors (green). 5HT1A auto-receptors are located on the somato-dendritic regions of serotonergic neurons and regulate serotonergic neuronal activity. The unused extracellular serotonin is re-uptake (black arrow) by presynaptic serotonin transporters (blue channel), to be recycled and reused. During inflammation (right panel, red lightning bolt), TNFα concentration increases (red lightning bolt). This reduces tryptophan (fewer orange discs in right vs. left hand panel) involved in serotonin synthesis (thinner black vertical arrows). Also, it leads to fewer synaptic vesicles containing serotonin (fewer black circles in presynaptic neuron). Less serotonin is released. Inflammation also causes serotonin transporters to rapidly reuptake the available extracellular serotonin (bold black arrow). Further, an increase in 5HT1A auto-receptors is observed. **(B)** Inflammation changes at a synaptic terminal in the subcallosal cingulate cortex (SCC). The postsynaptic receptor distribution is complementary to that of the PFC: the SCC has an abundance of inhibitory 5HT1A receptors (green cylinders) compared to the excitatory 5HT2A receptors (grey cylinders). Under inflammation (right panel), a reduction in serotonin is observed. In depression, a reduction in postsynaptic 5HT1A receptors and an increase in the 5HT1A auto-receptors is observed. **(C)** (Left panel) Excitatory (red) and inhibitory (yellow) neuronal populations and their connections. Excitatory currents are mediated by NMDA receptors. Inhibitory by GABA receptors. Brain activity is coupled with the neurotransmitter system. $I_{o}^{n}$ (black arrows) are external currents received from other cortical and subcortical brain regions. Serotonergic currents, $M_{5HT}^{n}$ are shown by blue arrows. The superscript n denotes the PFC and SCC brain regions. Inflammation causes a $M_{5HT}^{n}\;$ reduction. Right panel: PFC-SCC brain network. The two regions are coupled by long-range NMDA connections targeting excitatory populations. **(D)** Elevated TNFα concentration (red module) due to inflammation reduces serotonin (blue module) by impacting synthesis and reuptake. This, in turn, reduces serotonergic input to the PFC-SCC network (green module). Excitatory population activity within PFC and SCC reciprocally modulates the serotonin concentration (double arrow). Further, the PFC-SCC circuit receives — and sends — signals to many other regions in the brain, like hippocampus, thalamus and amygdala.


Here, n={PFC,SCC}  labels the PFC and SCC brain region. $r_{\left(E,I\right)}^{n}\;$ describes the excitatory (E) and inhibitory (I) neuronal population firing activity of the PFC and SCC brain regions. Within each brain region these neuronal populations are reciprocally connected to each other (**Figure 1B**, left panel). The neuronal response function $H_{\left(E,I\right)}$ (44) serves as an input-output function transforming the currents $I_{\left(E,I\right)}^{n}$ to produce $r_{\left(E,I\right)}^{n}$ . $I_{\left(E,I\right)}^{thr}$ defines the threshold current. Further, the parameters $g_{\left(E,I\right)}$ and $d_{\left(E,I\right)}$ define the gain factor for the slope and curvature of $H_{\left(E,I\right)}$ around $I_{\left(E,I\right)}^{thr}$ respectively.

(3) $$\begin{array}{c} I_{E}^{PFC}=\;w_{+}J_{NMDA}^{PFC}S_{E}^{PFC}-JS_{I}^{PFC}+\;W_{E}^{5HT,\;PFC}R^{PFC}M_{5HT}^{PFC}\\ +\;W_{E}^{PFC}I_{o}^{PFC}+J_{NMDA}^{PFC}C_{SCC-PFC}S_{E}^{SCC}\; \end{array}$$

(4) $$\begin{array}{c} \;I_{E}^{SCC}=\;w_{+}J_{NMDA}^{SCC}S_{E}^{SCC}-JS_{I}^{SCC}-\;W_{E}^{5HT,SCC}R^{SCC}M_{5HT}^{SCC}\\ +\;W_{E}^{SCC}I_{o}^{SCC}+\;J_{NMDA}^{SCC}C_{PFC-SCC}S_{E}^{PFC}\; \end{array}$$

(5) $$\begin{array}{c} \;I_{I}^{PFC}=\;J_{NMDA}^{PFC}S_{E}^{PFC}-S_{I}^{PFC}+\;W_{I}^{5HT,PFC}R^{PFC}M_{5HT}^{PFC}\\ +\;W_{I}^{PFC}I_{o}^{PFC}\;\;\;\;\;\; \end{array}$$

(6) $$\begin{array}{c} I_{I}^{SCC}=\;J_{NMDA}^{SCC}S_{E}^{SCC}-S_{I}^{SCC}-\;W_{I}^{5HT,SCC}R^{SCC}M_{5HT}^{SCC}\\ +\;W_{I}^{SCC}I_{o}^{SCC}\;\;\;\;\;\; \end{array}$$

The currents $I_{\left(E,I\right)}^{n}$ in Equations 3−6 include inputs from excitatory and inhibitory neuronal populations, serotonergic currents $M_{5HT}^{n}$ , and external currents $I_{O}^{n}$ . $I_{O}^{n}$ are scaled by the weights $W_{E}^{n}$ and $W_{I}^{n}\;$ for the E and I populations respectively. Further, $w_{+}\;$ refers to the recurrent excitation weight, $J_{NMDA}^{n}$ is the excitatory synaptic coupling and J is the local feedback synaptic coupling.

Both regions contain 5HT1A and 5HT2A receptors. PFC has an abundance of excitatory serotonergic 5HT2A receptors whereas the SCC is dominated by inhibitory serotonergic 5HT1A receptors (45) (**Figure 1A, B**). For simplicity, we considered contributions from 5HT2A receptors in PFC and 5HT1A receptors in SCC. This is described by the parameter $.R^{n}$ Also, $W_{\left(E,I\right)}^{5HT,n}$ weight the excitatory and inhibitory inputs from the serotonergic system. PFC and SCC interact through bidirectional long-range excitatory connections (**Figure 1C**, right panel). $C_{SCC-PFC}$ and $C_{PFC-SCC}$ are the coupling constants between these brain regions. Synaptic dynamics are modelled by the following Equations 7, 8:


(7) $$\frac{dS_{E}^{n}}{dt}=\;-\frac{S_{E}^{n}}{\tau_{NMDA}}+\;\left(1-\;S_{E}^{n}\right)\gamma r_{E}^{n}+\;\sigma v\left(t\right)\;\;\;$$

(8) $$\frac{dS_{I}^{n}}{dt}=\;-\frac{S_{I}^{n}}{\tau_{GABA}}+\;r_{I}^{n}+\;\sigma v\left(t\right)\;\;\;\;\;\;\;$$

The synaptic gating variables, $S_{\left(E,I\right)}^{n}\;$ depend on the time constants of NMDA, $\tau_{NMDA},\;$ and GABA, $\tau_{GABA},\;$ respectively.  v(t) is the uncorrelated standard Gaussian noise with an amplitude σ given by v(t)= N(0,1) and Cov(v(t),v(t'))=δ(t−t') . v(t) is drawn from a standard normal distribution with mean zero and unit variance. The value of v(t)  at a given time point *t* has no influence on its value at a later time point *t’.* The variability in v(t) stems from fluctuations in synaptic plasticity, driven by changes in the opening and closing of ligand-gated NMDA and GABA receptors at excitatory and inhibitory synapses. Additionally, trial-to-trial variability in responses to stimuli results from alterations in the internal states of neurons and the network. Moreover, numerous stochastic processes at the molecular level such as the diffusion and binding of signaling molecules to receptors, synaptic vesicle fusion, and the opening and closing of ion channels contributes further to this variability (46).

Serotonergic current, $M_{5HT}^{n},\;$ dynamics are given by Equation 9:


(9) $$\tau_{5HT}\frac{dM_{5HT}^{n}}{dt}=\;-M_{5HT}^{n}+\;\frac{J_{5HT}}{1+\;e^{-\beta \left(\left[5HT^{n}\right]+1\right)}}\;\;\;$$

The serotonin concentration appearing above changes over time as follows:


(10) $$\frac{d\left[5HT^{n}\right]}{dt}=\;\alpha C_{BR}^{n}r_{E}^{n}X_{1}-\;\frac{V_{max}\left[5HT^{n}\right]}{K_{m}+\;\left[5HT^{n}\right]}X_{2}\;\;\;\;$$

    where $X_{1}^{n}\;$ and $X_{2}^{n}$ are given by


(11) $$X_{1}^{n}=\frac{c_{1,n}^{m}}{c_{1,n}^{m}+\theta \left(\left[Cyt\right]-{\left[Cyt\right]}_{b}\right)*{\left(\left[Cyt\right]/{\left[Cyt\right]}_{b}\right)}^{m}}\;\;$$

(12) $$X_{2}^{n}=1+c_{2,n}\theta \left(\left[Cyt\right]-{\left[Cyt\right]}_{b}\right)*[Cyt]/{\left[Cyt\right]}_{b}\;\;$$

We explain below the parameters that appear above. Serotonin concentration $\left[5HT^{n}\right],$ and its dynamics following synthesis and reuptake are modelled by Equation 10. This is a Michaelis-Menten kinetic scheme, where α controls the serotonergic current so that the drive in Equation 9 is around the center of the sigmoid. $C_{BR}^{n}$ is the fiber density connectivity between the prefrontal cortex (SCC) and the raphe nuclei. $V_{max}$ and $K_{m}$ are the Michaelis-Menten constants that define the maximum re-uptake rate and the serotonin concentration at which the re-uptake rate is half of the maximum rate respectively.

The expression $\left[Cyt\right]/{\left[Cyt\right]}_{b}$ is the degree of inflammation. ${\left[Cyt\right]}_{b}$ is the basal mean TNFα cytokine concentration in controls and [Cyt] is the elevated mean TNFα cytokine level under inflammation. The degree of inflammation takes only positive values, see Results and (36) for details. In this study, we have focused on group means, rather than individual variability, of the TNFα levels from (36). In our present framework, it is not possible to provide continuous intervals for the degree of inflammation as the study (36) that we used to quantify the cytokine TNFα values associated with depression obtained overlapping intervals of TNFα under mild, moderate and severe depression conditions. The term $X_{1}^{n}\;$ in Equation 10 models the cytokine effects on serotonin synthesis as a result of reduced tryptophan $.\;c_{1,n}\;$ and m defines the shape and steepness of $X_{1}^{n}$ . The term $X_{2}^{n}\;$ in Equation 10 describes the rapid increase in serotonin re-uptake. $c_{2,n}$ describes the increased re-uptake rate. $\tau_{5HT},\;J_{5HT}$ and β are the parameters that define the time constant, range and slope of the serotonergic currents. In Equations 11, 12, θ represents the Heaviside function. This function takes the value one when [Cyt] exceeds ${\left[Cyt\right]}_{b},\;$ else it takes a value of zero.

### 2.1 Modelling drug treatments

In this study, various drug treatments were modelled by the following parameter changes.

1. **SSRI treatment:** The value of $K_{m}$ in Equation 10 is varied from 170nM to 200nM.

2. **Anti-inflammatory treatment:** The parameter *B* in Equations 13, 14. models vs. anti-inflammatory blocker effects (B= 0.55). Here, θ is the Heaviside Function.


(13) $$\;\;\;\;\;\;\;X_{1}^{n}=\frac{c_{1,n}^{m}}{c_{1,n}^{m}+\theta \left(\left[Cyt\right]-{\left[Cyt\right]}_{b}\right)*B*{\left(\left[Cyt\right]/{\left[Cyt\right]}_{b}\right)}^{m}}\;$$

(14) $$X_{2}^{n}=1+c_{2,n}\theta \left(\left[Cyt\right]-{\left[Cyt\right]}_{b}\right)*B*\left[Cyt\right]/{\left[Cyt\right]}_{b}\;$$

3. **Co-administration of SSRIs and anti-inflammatory drug treatment:** Dual treatment is implemented by simultaneously changing $K_{m}$ to 200nM and B to 0.55.

All simulations were performed using MATLAB. The differential equations were solved numerically using the Euler-Maruyama method with a time step of 0.1ms. Simulations ran for 7 seconds of simulated time (47). Our simulations started from random initial conditions. The activity reached a steady state after discarding an initial transient of 1 second. We have chosen 7 seconds for convenience similar to (47–49). We have followed standard practice while simulating neural mass equations, where the number of repetitions is normally taken as n =100 (47, 50, 51).

This data was then used to obtain the mean and sem values. The values of the various parameters used in the simulations along with their descriptions are provided in **Tables 1**, **2**.

**Table 1:** Summary of the Neuronal model parameters.

Parameters	PFC subnetwork	SCC subnetwork	Description	Sources
$I_{E}^{thr}$	0.4nA	0.4nA	Threshold currents for excitatory pool of neurons	(a)
$I_{I}^{thr}$	0.286nA	0.286nA	Threshold currents for inhibitory pool of neurons	(a)
$I_{o}^{n}$	0.32nA	0.45nA	Overall effective external input	(c)
$g_{E}$	310nC^-1^	310nC^-1^	Gain factor for the slope of $H_{E}$ around $I_{E}^{thr}$	(a)
$g_{I}$	615nC^-1^	615nC^-1^	Gain factor for the slope of $H_{I}$ around $I_{I}^{thr}$	(a)
$d_{E}$	0.16	0.16	Gain factor for the curvature of $H_{E}$ around $I_{E}^{thr}$	(a)
$d_{I}$	0.087	0.087	Gain factor for the curvature of $H_{I}$ around $I_{I}^{thr}$	(a)
$J_{NMDA}^{n}$	0.15nA	0.33nA	Excitatory synaptic coupling	(a), (c) for the SCC network
J	1.135	1.135	Local feedback synaptic coupling	(c)
$W_{E}^{5HT,\;n}$	0.48	0.19	Weight of the excitatory inputs of the serotonergic system	(a), (c) for the SCC network
$W_{I}^{5HT,n}$	0.47	0.19	Weight of the inhibitory inputs of the serotonergic system	(a), (c) for the SCC network
$W_{E}^{n}$	1	1	Weight for the excitatory populations	(a)
$W_{I}^{n}$	0.7	0.8	Weight for the inhibitory populations	(a)
$w_{+}$	1.4	1.4	Recurrent excitation weight	(a)
$R^{n}$	0.6	0.6	Density of serotonergic receptors – 5HT2A for PFC and 5HT1A for the SCC	(a), (b) for the SCC network
$\tau_{NMDA}$	100ms	100ms	Time constants of NMDA	(a)
$\tau_{GABA}$	10ms	10ms	Time constants of GABA	(a)
γ	0.641*10^-3^	0.641*10^-3^	NMDA kinetic parameter	(a)
$C_{SCC-PFC}$	–	0.01	Coupling constant between SSC and PFC brain regions	(a)
$C_{PFC-SCC}$	0.005	–	Coupling constant between PFC and SCC brain regions	(a)
σ	0.01nA	0.01nA	Amplitude of the Gaussian noise	(a)

Here, the superscript label *n* in the parameters refers to PFC in Column 2 and SCC in Column 3. The values of these parameters have been taken from sources indicated below:

(a) for values taken from (27): Kringelbach ML, Cruzat J, Cabral J, Knudsen GM, Carhart-Harris R, Whybrow PC et al. Dynamic coupling of whole-brain neuronal and neurotransmitter systems. Proc Natl Acad Sci USA. (2020) 117: 9566-9576.

(b) for values chosen somewhat arbitrarily without targeted optimization.

(c) for values optimized over several preliminary simulations.

**Table 2:** List of parameters for serotonergic inputs.

Parameters	PFC subnetwork	SCC subnetwork	Description	Source
α	5	5	Serotonergic current so that the drive in Equation 9 is around the center of the sigmoid	(a)
$C_{BR}$	15	15	Fiber density connectivity between the prefrontal cortex (SCC) and the raphe nuclei	(a)
$V_{max}$	1300nMs^-1^	1300nMs^-1^	Michaelis-Menten constant for the maximum re-uptake rate	(a)
$K_{m}$	170 (Control condition) 200 (SSRI treatment)	170 (Control condition) 200 (SSRI treatment)	Michaelis-Menten constant at which the serotonin concentration re-uptake rate is half of the maximum rate	(a),(b)
$c_{1,n}$	5	4	Cytokine impact modelled on Serotonin synthesis	(e)
m	2	2	Cytokine impact modelled on Serotonin synthesis	(e)
$c_{2,n}$	0.019	0.019	Serotonin re-uptake rate	(e)
$\tau_{5HT}$	120ms	120ms	Time constant of the serotonergic currents	(a)
$J_{5HT}$	1	1	Range of the serotonergic currents	(a)
β	0.008	0.008	Slope of the serotonergic currents.	(a)
$\frac{\left[Cyt\right]}{{\left[Cyt\right]}_{b}}$	1 (Control condition) 1.25 (Mild inflammation) 1.4 (Moderate inflammation) 2.3 (Severe inflammation)	1 (Control condition) 1.25 (Mild inflammation) 1.4 (Moderate inflammation) 2.3 (Severe inflammation)	Degree of inflammation	(c)
B	0.55	0.55	Anti-inflammatory treatment	(e)

The superscript label *n* in the parameters refers to PFC in Column 2 and SCC in Column 3. The values of these parameters have been taken from sources indicated below:

(a) for values taken from (27): Kringelbach ML, Cruzat J, Cabral J, Knudsen GM, Carhart-Harris R, Whybrow PC et al. Dynamic coupling of whole-brain neuronal and neurotransmitter systems. Proc Natl Acad Sci USA. (2020) 117: 9566-9576

(b) for values taken from (65): John CE, Jones SR. Voltammetric characterization of the effect of monoamine uptake inhibitors and releasers on dopamine and serotonin uptake in mouse caudate-putamen and substantia nigra slices. Neuropharmacology. (2007) 52:1596-1605. doi: 10.1016/j.neuropharm.2007.03.004.

(c) for values taken from (36): Zou W, Feng R, Yang Y. Changes in the serum levels of inflammatory cytokines in antidepressant drug-naïve patients with major depression. PLoS One (2018) 13: e0197267. doi: 10.1371/journal.pone.0197267

(d) for values chosen somewhat arbitrarily without targeted optimization.

(e) for values optimized over several preliminary simulations.

## 3 Results

### 3.1 A computational model of inflammation-mediated changes of serotonergic availability

Our model links inflammation, the serotonergic system and brain activity. It describes how inflammation alters serotonergic currents and ensuing brain activity. The effect of inflammation on serotonin is two-fold. This is shown in **Figures 1A, B**, see also (31, 52). (i) Reduction of serotonin synthesis: cytokines stimulate the production of an enzyme called indoleamine 2,3-dioxygenase (IDO). This directs tryptophan, a precursor of serotonin, into the kynurenine pathway: tryptophan produces more kynurenine and other metabolites, instead of serotonin (53). (ii) Increase of serotonin reuptake: the enzyme IDO increases the activity of serotonin transporters. These are proteins that clear serotonin from the extracellular medium by transporting it back into the presynaptic terminal (15).

Our model describes how TNFα concentration affects brain activity; specifically, in the cingulo-frontal circuit thought to underlie depression (**Figure 1**). It rests on the premise that depression is a circuit disorder (4, 9, 54) affecting the frontal cortex, insula, thalamus and other areas. Among them, the circuit modelled here shows highly consistent depression-related abnormalities and is the target of treatments including medication, psilocybin treatments and psychotherapy (43). This circuit includes PFC and SCC is also the target of Deep brain stimulation (DBS) (8). Transcranial magnetic stimulation (TMS) impacts this circuit—changing the balance of neural activity between and finding the spot with maximal anti-correlation of the SCC and PFC (55).

To our knowledge, the model described in this foundational paper is the first to address how changes in cytokine concentration affect neural activity. The neural mass model includes excitatory and inhibitory neuronal populations driven by excitatory NMDA currents (shown in red, **Figure 1C**, left panel), inhibitory GABA currents (yellow) and external input $I_{o}\;$ (black), see also (28) who described glutamate dysregulation and its effects on treatment response and EEG rhythms. Additionally, our model includes serotonergic currents $M_{5HT}$ (blue), whose amplitude is determined by the extracellular serotonin concentration. This concentration is modulated by changes in serotonin synthesis and reuptake due to inflammation (*Methods*) (27). A novel feature of our model is the dependence of serotonin synthesis and reuptake on changes in cytokine concentration due to elevated TNFα levels of the sort observed in inflammation. The following section explains this dependence in detail.

Serotonin synthesis (release) depends on the density of the fibers connecting the DRN with SCC or PFC. It also depends on the activity of the excitatory neuronal population in SCC or PFC. The excitatory long-range reciprocal interaction between the PFC and SCC is depicted in **Figure 1C** (right panel). In general, receptor expression varies systematically across cortical areas (56). Among all receptors, serotonin shares the most marked change (gradient) in expression over the cortex. This is also the case in the regions considered here. PFC has an abundance of excitatory 5HT2A receptors (**Figure 1A**), while SCC has inhibitory 5HT1A receptors (**Figure 1B**) (45). Both the brain regions have 5HT1A and 5HT2A serotonergic receptors. The receptor densities of 5HT1A in the PFC and 5HT2A in the SCC are low (**Figures 1A, B**). Thus, we modelled contributions of 5HT2A receptors in PFC and 5HT1A receptors in SCC. The model parameter $R^{n}$ expressing receptor densities were based on measurements with positron emission tomography (PET) (27). Inflammation parameters were chosen to obtain serotonin levels reported in depression studies. These and other model parameters can be found in **Tables 1**, **2**. They follow (27, 36). Elevated levels of TNFα (red box in **Figure 1D**) cause a reduced synthesis and increased reuptake of extracellular serotonin (blue box). This, in turn, alters activity in the cingulo-frontal brain network (green box in **Figure 1D**).

### 3.2 Inflammation leads to cytokine increase and reduced serotonin

Inflammation increases the concentration of the cytokine TNFα. We first asked how this affects extracellular serotonin levels in PFC and SCC. TNFα increase stimulates the enzyme IDO that drives tryptophan into producing kynurenine and impairs serotonin synthesis. IDO also increases serotonin reuptake. Our model parametrizes the change in TNFα concentration as a result of inflammation (see *Methods* and below). It also predicts the corresponding changes in serotonin synthesis and reuptake that result from TNFα changes.

**Figure 2:**
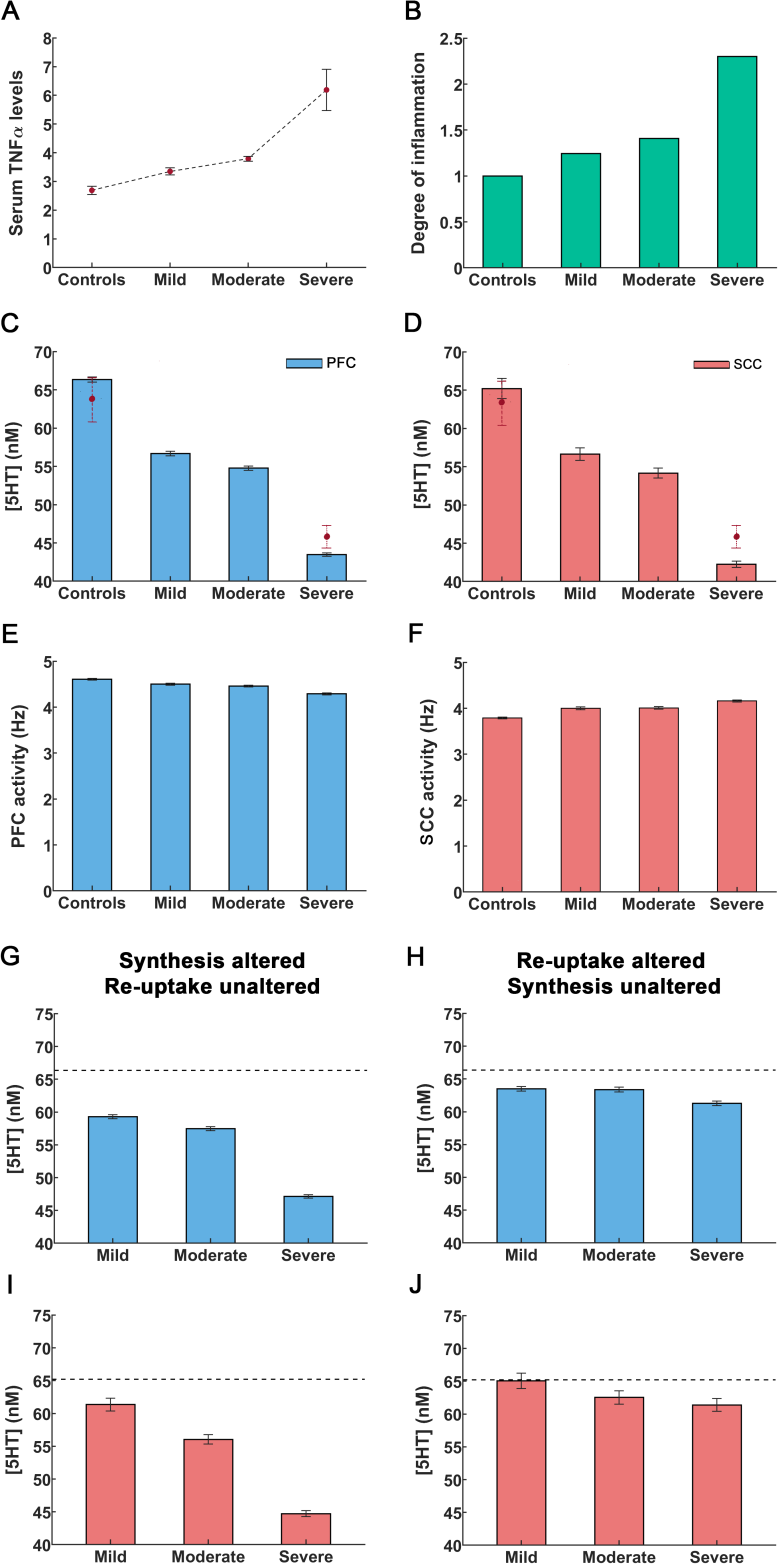
An increase in cytokines caused by peripheral inflammation leads to reduction in serotonin concentration and alterations in cingulo-frontal network activity. **(A)** Cytokine TNFα concentrations in controls are denoted by ${\left[Cyt\right]}_{b}.\;$ Concentrations in depressed individuals by [Cyt] . The solid circles refer to mean TNFα concentrations and errors bars are the sem values taken from Zou et al. (36). **(B)** Using concentrations from [A], we constructed the concentration ratio $\left[Cyt\right]/{\left[Cyt\right]}_{b}$ .This is referred to as the degree of inflammation. The control condition corresponds to a degree of inflammation of one (leftmost bar). Other bars denote the degree corresponding to mild, moderate and severe inflammation as defined in Zou et al. (36). **(C, D)** Rise in inflammation degree reduces serotonin concentrations in PFC and SCC. The solid circles with dashed lines depict serotonin concentrations observed in controls and in inflammation-induced severe depression, as reported by Hersey et al. (57). **(E, F)** Rise in inflammation degree reduces theta band activity. **(G, I)** The extracellular serotonin levels in the PFC and SCC assuming inflammation affects serotonin synthesis only (cf. *X_1_* term in Equations 10 in *Methods*) and reuptake is unaltered. **(H, J)** Extracellular serotonin levels assuming inflammation affects serotonin reuptake only (cf. *X_2_* term in Equations 10) and synthesis is unaltered. The black dash line in the figures G-I refer to the extracellular serotonin levels in controls. The error bars refer to the sem values obtained from 100 simulations run for each condition.

We characterized the increase in TNFα as the ratio $\left[Cyt\right]/{\left[Cyt\right]}_{b}$ . We call this, the degree of inflammation. It describes the relative increase in the TNFα cytokine concentration [Cyt] relative to controls, denoted by ${\left[Cyt\right]}_{b}$ . ${\left[Cyt\right]}_{b}$ is equal to 2.69 ± 0.14pg/mL (mean ± sem) as measured in human serum (36), **Figure 2A**. We considered a graded rise in the degree of inflammation. Our analysis followed (36). These authors used the Hamilton Depression Rating Scale (HAMD) scores and found linear correlations between depression severity and TNFα concentration. Similar to Zou et al. (36), we considered three inflammation conditions: mild, moderate and severe (**Figure 2B**). A 1.25-fold rise in inflammation degree corresponds to mild inflammation where [Cyt] was 3.35 ± 0.13pg/mL. A 1.4-fold rise corresponds to moderate inflammation and a 2.3-fold rise to severe inflammation. The corresponding cytokine concentrations  [Cyt] were 3.79 ± 0.08pg/mL and 6.19 ± 0.72pg/mL respectively (36) [**Figure 2A**, two rightmost bars].

Our model, predicts the extracellular serotonin concentration for different degrees of inflammation. Serotonin concentration for controls ( $\frac{\left[Cyt\right]}{{\left[Cyt\right]}_{b}}=1)$ was predicted to be 66.35 ± 0.35nM in PFC (**Figure 2C**, leftmost bar) and 65.21 ± 1.32nM in SCC (**Figure 2D**, leftmost bar). These concentrations are similar to those reported in (57, see below). In severe depression, a 2.3-fold increase in the degree of inflammation led to a serotonin concentration of 43.48 ± 0.22nM in PFC and 42.25 ± 0.40nM in SCC (57) (**Figures 2C, D**, rightmost bars). In mild inflammation concentration was 56.69 ± 0.29nM in PFC and 56.63 ± 0.82nM in SCC. Last, a 1.4-fold rise in the degree of inflammation (moderate) rendered the concentration 54.77 ± 0.28nM in PFC and 54.16 ± 0.66nM in SCC (**Figures 2C, D**). Serotonin concentrations for the control and severe inflammation conditions are similar to 63.68 ± 3nM and 46.91 ± 1.48nM reported in (57). Our model also predicted concentrations for mild and moderate depression considered in (36). These are novel results. To the best of our knowledge no experimental data exists for these conditions. An important point to note here is that the authors in (36) report measurements in mice. As concentrations are normalized to unit volume, we assumed that changes from the control condition for different levels of inflammation will be similar across species. To produce inflammation and induce depression-like behavior (57) administered lipopolysaccharide (LPS), a known immune system stimulator.

Next, we distinguished inflammation effects on serotonin synthesis and reuptake. These were quantified by the *X_1_* and *X_2_* terms in our model (Equations 10 in *Methods*). Reduced synthesis was described by the *X_1_* term, while elevated reuptake rates entailed by the *X_2_* term. These terms capture differences due to excitatory 5HT2A receptor densities in PFC and inhibitory 5HT1A receptor densities in SCC. We then considered the effect of changing one of these terms and keeping the other constant. Assuming serotonin synthesis was altered without TNFα affecting reuptake (i.e., the *X_1_* term) led to a significant reduction in the extracellular serotonin levels (**Figures 2G, I**). These reductions were similar to those obtained above when assuming that TNFα affected both synthesis and reuptake (**Figures 2C, D**). Assuming that only serotonin reuptake was impacted by TNFα (*X_2_* term) led to a slight reduction in serotonin levels (**Figures 2H, J**). Thus, changes in serotonin concentration due to inflammation seem to be driven by TNFα effects on synthesis not reuptake.

### 3.3 Dysregulation in cingulo-frontal network activity as a result of inflammation

We then turned to brain activity. Our model generates neuronal activity in PFC and SCC. Both are main hubs in a medial prefrontal network known to mediate chronic stress and depression (58). We considered control responses and responses for different degrees of inflammation as above. For controls, resting state PFC activity was 4.61 ± 0.02Hz (**Figure 2E**, leftmost bar), similar to observed DLPFC monkey recordings of 5Hz and 6Hz reported by (59, 60). The model predicted reduced PFC neural activity for mild and moderate inflammation i.e. 4.50 ± 0.02Hz and 4.46 ± 0.02Hz respectively (**Figure 2E**, middle bars). Under severe inflammation, activity was reduced to 4.29 ± 0.02Hz. This amounts to about 7% reduction in activity between control and severe inflammation conditions (**Figure 2E**, rightmost bar, see *Discussion*), similar to recordings by (61, 62).

Our model also predicted that SCC theta band activity increased when the degree of inflammation increased. Activity in controls was found to peak at 3.79 ± 0.02Hz (**Figure 2F**, leftmost bar). Similar predictions for mild and moderate inflammation included peaks at 4.00 ± 0.03Hz and 4.01 ± 0.03Hz respectively (**Figure 2F**, middle bars). For severe inflammation, activity was found to peak at 4.16 ± 0.02Hz (**Figure 2F**, rightmost bar). This amounts to a 10% increase in SCC network activity compared to controls (see *Discussion*), similar to EEG recordings by (63) who report an increase in theta band activity of about 5%.

Overall, inflammation had opposite effects in PFC and SCC see also (64). This is explained by the difference of serotonergic receptors in these regions. PFC has an abundance of excitatory 5HT2A receptors, while SCC has an abundance of inhibitory 5HT1A receptors. Thus, our model found that frontal activity was reduced with inflammation, while limbic activity increased. Crucially, it predicted subtle changes and their dependence on the degree of inflammation that matched experimental recordings.

### 3.4 SSRIs alleviate serotonin deficiency under mild inflammation

**Figure 3:**
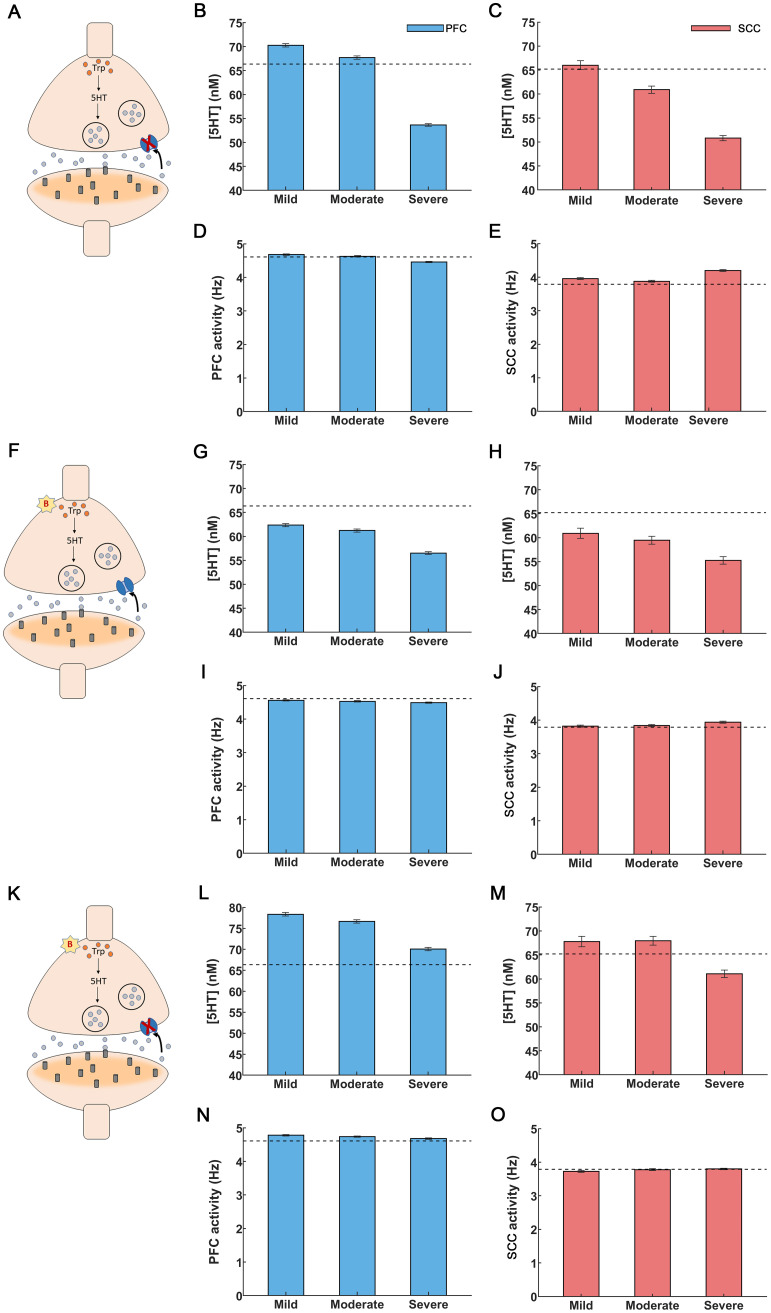
Drug interventions for inflammation-induced depression. **(A-E)** SSRI treatment. **(A)** A synapse under depression: serotonin synthesis is decreased and reuptake is increased, cf. (**Figure 1A**). SSRI application blocked serotonin transporters shown as a red “X”. **(B)** Administration of SSRIs led to a rise in PFC serotonin levels. SSRIs restored serotonin levels for mild and moderate inflammation but failed to do so for severe inflammation. **(C)** SCC serotonin was restored to control levels only for mild inflammation. **(D)** PFC activity was restored to control levels for mild and moderate inflammation. **(E)** Elevation in SCC activity was alleviated for mild and moderate inflammation. **(F-J)** Anti-inflammatory treatment. **(F)** Anti-inflammatory drugs caused a reduction in cytokines, depicted by a red “B” **(G, H)** Anti-inflammatory drugs raised serotonin levels but were insufficient to fully restore them to control levels for PFC and SCC, regardless of the degree of inflammation. **(I,J)** PFC and SCC activity were restored to control levels (PFC activity increased and SCC activity decreased). **(K-O)** Co-administration of SSRIs and anti-inflammatory treatment. **(K)** Synapse in depression undergoing simultaneous treatment with SSRIs (red “X”) and anti-inflammatory drugs (red “B”). **(L)** Extracellular serotonin levels were restored in the PFC for all inflammation conditions. **(M)** SCC serotonin levels were restored for mild and moderate inflammation. **(N, O)** Co-administration of SSRIs and anti-inflammatory drugs brought back PFC and SCC activity to control levels for all the inflammation conditions. The light blue (light red) bars in the figures depict extracellular serotonin and activity levels for the PFC (SCC) brain region. The black dashed lines refer to extracellular serotonin levels and activity in controls for PFC and SCC brain regions. The error bars refer to the sem values obtained from 100 simulations run for each condition.

Next, we considered drug treatments, and their limitations. We first modelled SSRI effects. In the present framework, SSRIs correspond to a 10mg/kg dose of the SSRI escitalopram (57). We asked whether SSRIs could alleviate depression in the presence of inflammation. SSRIs are commonly used as the first line of treatment for depression. In our model, their administration (reuptake inhibition, red “X” in **Figure 3A**) is characterized using the Michaelis-Menten constant *K_m_*. Serotonin reuptake followed the Michaelis-Menten kinetics. The constant *K_m_* describes the binding affinity (or likeness) of serotonin to its transporter. A large *K_m_* corresponds to small affinity (denominator in Equations 10, see *Methods*). In the presence of a reuptake inhibitor the affinity of serotonin to its transporter is decreased (65). We therefore modelled SSRI effects by an increased value of *K_m_* (66).

We varied *K_m_* between 170 (controls) to 200, similar to fast scan cyclic voltammetry experiments by (65, 66). In that earlier work, serotonin concentration was measured in real time. The resulting estimates were fitted to the Michaelis-Menten based kinetic model and the constants $V_{max}$ and *K_m_* were obtained *(Methods)*. Caution was exercised in raising *K_m_* beyond 200nM, as this could lead to a rapid surge of serotonin in PFC and SCC, as observed in (65, 66), potentially leading to serotonin syndrome (67). Our model predicted that SSRI administration increases PFC and SCC serotonin concentration (**Figures 3B,C**). For mild and moderate inflammation, PFC serotonin concentration was 70.26 ± 0.36nM and 67.72 ± 0.35nM respectively (**Figure 3B**, leftmost and middle bars). Interestingly, the latter value (moderate inflammation) is close to control levels, i.e. 66.35 ± 0.35nM (shown by a dash line in **Figure 3B**, see also **Figure 2C** and (57)). However, for severe inflammation serotonin concentration was 53.63 ± 0.26nM (**Figure 3B**, rightmost bar). In other words, SSRI administration failed to restore serotonin to control levels. In SCC, our model predicted a serotonin concentration of 66.01 ± 0.93nM, 60.92 ± 0.76nM and 50.80 ± 0.55nM under mild, moderate and severe inflammation (**Figure 3C**). SSRIs restored serotonin levels to control levels (65.21 ± 1.32nM, shown by a dashed line in **Figure 3C**) for mild inflammation only—not moderate nor severe. This is distinct from PFC, where SSRIs restore serotonin levels to control conditions, for mild and moderate inflammation.

We then assessed the impact of SSRIs on brain activity. For mild and moderate inflammation, SSRI administration resulted in an increased PFC activity of 4.68 ± 0.02Hz and 4.63 ± 0.02Hz respectively. These values are close to control levels (4.61 ± 0.02Hz, **Figure 3D**) (60, 68),. Further, SSRI administration caused a reduction in SCC activity to 3.96 ± 0.03Hz and 3.88 ± 0.03Hz for mild and moderate inflammation. This is also similar to control activity (3.79 ± 0.02Hz, **Figure 3E**) (68). However, for severe inflammation SSRI treatment led to a PFC activity of 4.46 ± 0.02Hz and SCC activity of 4.21 ± 0.03Hz (**Figures 3D, E**, rightmost bars). Thus, SSRIs cannot restore brain activity to control levels in severe inflammation, which could be explained by the failure to restore serotonin concentration found above. This suggests that besides SSRIs, alternative treatments like targeting inflammation can be considered. This is what we did next. We studied anti-inflammatory drug effects on serotonin and brain activity.

### 3.5 Anti-inflammatory drugs restore impaired brain activity but not serotonin deficiency

Above, we saw that SSRIs do not alleviate serotonin deficiency in the cingulo-frontal network under severe inflammation. This could be due to elevated cytokine levels that are not reduced by merely blocking serotonin reuptake. We thus asked if anti-inflammatory drugs could remedy serotonin deficiency and impaired brain activity.

In our model, anti-inflammatory drug administration was modelled through changing the value of the parameter *B* (*Methods*). This parameter characterizes the percentage reduction of cytokine concentration that affects both serotonin synthesis and reuptake (Equations 13, 14 in *Methods*, red “B” in **Figure 3F**) and corresponds to the effect of the 20mg/kg dose of the anti-inflammatory drug, α-fluoromethylhistidine dihydrochloride (FMH) (57). We chose the parameter *B* (*B=*0.55), so that PFC serotonin levels rise to control levels of 63.68 ± 3nM when SSRIs and anti-inflammatory drugs are co-administered; the value found in (57, 69, 70). This is discussed in the next section. Here, we consider the effect of anti-inflammatory drugs only. Increasing the parameter value B results in a surge in serotonin, which may have long-term adverse effects, including serotonin syndrome and cardiovascular risks (67, 71). Our model predicted that for mild inflammation, anti-inflammatory drug administration increased cortical serotonin level to 62.35 ± 0.32nM (leftmost bar in **Figure 3G**), away from control levels of 66.35 ± 0.35nM (dash line in **Figure 3G**) (57). Further, for moderate and severe inflammation our model predicted serotonin levels to be 61.23 ± 0.33nM and 56.53 ± 0.29nM. Therefore, anti-inflammatory treatment caused an increase in serotonin levels but failed by itself to fully restore PFC serotonin to control levels regardless of inflammation degree. A similar response to anti-inflammatory drugs was found for SCC. Serotonin levels were only partially restored: 60.91 ± 1.08nM, 59.45 ± 0.83nM and 55.24 ± 0.77nM for mild, moderate and severe inflammation respectively. These are also away from control levels, shown by a dash line in (**Figure 3H**).

The results for brain activity were different. Interestingly, administration of anti-inflammatory drugs restored impaired neural activity to control levels for all degrees of inflammation and in both brain areas. Estimates (bars) in Figures (**Figures 3I, J**) almost overlap with control levels (shown with dash line, **Figures 3I, J**). Specifically, following anti-inflammatory drugs administration PFC activity was restored to 4.56 ± 0.02Hz (mild inflammation) and 4.53 ± 0.02Hz (moderate). Similarly, SCC activity to 3.82 ± 0.03Hz (mild) and 3.84 ± 0.03Hz (moderate). For severe inflammation, the corresponding values were 4.49 ± 0.02Hz (PFC, rightmost bar in **Figure 3I**) and 3.94 ± 0.03Hz (SCC, rightmost bar in **Figure 3J**).

In brief, administering anti-inflammatory drugs did not restore serotonin concentration to control levels. It seems, however, that the new concentrations were able to change gating dynamics and input currents and restore brain activity. This could not be achieved using SSRIs. This also points towards combined pharmacological treatments, to which we turned next.

### 3.6 Simultaneous SSRI and anti-inflammatory drug administration fully restored serotonin concentration to control levels

The foregoing numerical studies suggest that administering SSRIs or anti-inflammatory drugs on their own did not restore serotonin to control levels. Thus, we next simulated the effect of co-administering both drugs. We set the corresponding parameters in our model to treatment values, that is, *K_m_* to 200 and *B* to 0.55. Recall that *K_m_* reflects the rate of serotonin reuptake that is reduced by SSRI application. Further, *B* quantifies the percentage reduction in cytokine concentration as a result of anti-inflammatory drugs. Co-administering them led to an increase in serotonin concentration. Serotonin reuptake was reduced, and serotonin synthesis increased (red “B” and red “X” in **Figure 3K**). For severe inflammation, PFC serotonin levels were higher than control levels (66.35 ± 0.35nM) and had a value equal to 70.07 ± 0.39nM (**Figure 3L**, rightmost bar). Similarly, for SCC, the corresponding serotonin levels were 61.06 ± 0.77nM similar to control levels of 65.21 ± 1.32nM (**Figure 3M**, rightmost bar). Briefly, an increase in serotonin levels regardless of the severity of inflammation was observed. These results suggest that a joint treatment using SSRIs and anti-inflammatory drugs can remedy serotonin deficiency similar to observations by (69, 70, 72) where co-administration of SSRIs and anti-inflammatory drugs produced serotonin levels of 87.91 ± 2.72 nM.

Co-administering SSRIs and anti-inflammatory drugs also restored brain activity across all degrees of inflammation (**Figures 3N, O**). This could be driven by anti-inflammatory drugs that were found earlier to restore activity to control levels when administered on their own. In the case of dual administration, — for the case of severe inflammation — the PFC activity was 4.68 ± 0.02Hz and SCC activity was 3.80 ± 0.02Hz (**Figures 3N, O**, rightmost bars). These values were similar to control levels: 4.61 ± 0.02Hz and 3.79 ± 0.02Hz in PFC and SCC respectively (59, 60) (**Figures 2E, F**, leftmost bar). In summary, we found that anti-inflammatory treatment together with SSRIs restored both network activity and neurotransmitter function.

In a separate set of analyses, we considered another effect of inflammation: besides serotonin, inflammation affects NMDA receptors and changes glutamate levels. This is due to degradation of tryptophan through the kynurenine pathway. Tryptophan degradation increases kynurenine metabolites (73, 74). One of them, quinolinic acid, is an NMDA receptor agonist that also increases extracellular glutamate (75). It blocks glutamate reuptake through astrocytes by reducing amino acid transporter 2 (EAAT2) (76, 77). This is important in depression, as elevated levels of quinolinic acid have been associated with inflammation and suicidal attempts (73). NMDA receptor activation of the sort induced by inflammation is known to play a role in depression. Depression symptoms were reduced following the administration of ketamine (an NMDA receptor antagonist) (73). Also, experimental findings by Walker et al. (78) demonstrated that inflammation-induced depression is mediated by NMDA receptors. To model these effects, we increased the NMDA time constant by 5% in SCC (79), see also (28, 80).

Separate administration of SSRIs and anti-inflammatory drugs did not restore control serotonin and brain activity levels. Our model predicted a reduction in serotonin and an increase in SCC activity after increasing the NMDA receptor time constant (**Supplementary Figures 1A, B**). SSRI administration reversed these effects on serotonin in SCC but failed to restore control function (**Supplementary Figure 1C**). SCC serotonin concentration was 63.34 ± 0.82nM, 62.19 ± 0.84nM and 50.15 ± 0.56nM for mild, moderate and severe inflammation respectively (control levels were 65.21 ± 1.32nM, shown using dash lines in **Supplementary Figure 1C**). SCC activity was restored to control levels for mild inflammation (3.79 ± 0.02Hz shown through dash lines in **Supplementary Figure 1D**) but not for moderate and severe inflammation. Activity levels for mild, moderate and severe inflammation were 3.87 ± 0.02Hz, 3.94 ± 0.03Hz and 4.18 ± 0.03Hz respectively.

Anti-inflammatory drugs in isolation also led to a serotonin increase but failed to achieve control concentrations (**Supplementary Figure 1E**). SCC serotonin concentrations were found to be 61.41 ± 0.89nM, 61.63 ± 1.02nM and 55.02 ± 0.77nM for mild, moderate and severe inflammation respectively. Further, SCC activity was restored to control levels (**Supplementary Figure 1F**). It was 3.87 ± 0.03Hz, 3.90 ± 0.03Hz and 3.97 ± 0.03Hz for mild, moderate and severe inflammation respectively. Similarly, to the results above, the control state was restored using a combined pharmacological treatment. Co-administration of SSRIs and anti-inflammatory drugs led to a complete restoration of serotonin concentrations: 74.84 ± 1.28nM, 69.46 ± 0.94nM and 63.55 ± 0.86nM (**Supplementary Figure 1G**). SCC activity levels were also restored: 3.91 ± 0.03Hz, 3.82 ± 0.03Hz and 3.91 ± 0.02Hz.

### 3.7 Reduced postsynaptic receptor density as a mechanism to cope with depression

The preceding analyses focused primarily on drug effects on presynaptic neurons. Newer drugs target postsynaptic neurons. Postsynaptic 5HT1A receptor density in SCC has been found to be reduced in depressed individuals (81, 82). Thus, in the last set of analyses, we used our model to predict changes in serotonin concentration and SCC activity for reduced postsynaptic receptor density.

We quantified postsynaptic receptor density by a parameter *R* and reduced this parameter by about 8% in accordance with (83). This change left PFC serotonin levels unaffected (not shown). SCC serotonin levels for mild, moderate and severe inflammation were 66.64 ± 0.96nM, 62.18 ± 0.81nM and 49.16 ± 0.48nM (**Supplementary Figure 2A**). They were higher than the corresponding values of 56.63 ± 0.82nM,54.16 ± 0.66nM and 42.25 ± 0.40nM when receptor changes were not included (**Figure 2D**). Thus, reducing postsynaptic receptor density could act as a compensatory mechanism used by the SCC to cope with serotonin deficiency. At the same time, SCC hyperactivity persisted. SCC activity was 4.40 ± 0.03Hz, 4.38 ± 0.02Hz and 4.66 ± 0.03Hz for mild, moderate and severe inflammation respectively (**Supplementary Figure 2B**).

We then asked what the effect of a simultaneous SSRI treatment would be. Following SSRI administration, serotonin rose above control levels for mild and moderate inflammation: 76.11 ± 1.13nM and 73.35 ± 1.02nM (control levels were 65.21 ± 1.32nM and are shown by dashed lines in **Supplementary Figure 1C**). As with the previous results (that did not consider reduced postsynaptic receptor density, **Figure 3B**), this was not the case for severe inflammation. Our model predicted a serotonin concentration of 55.37 ± 0.60nM for severe inflammation. The corresponding SCC activity levels were 4.39 ± 0.03Hz, 4.36 ± 0.03Hz and 4.50 ± 0.03Hz, well above control levels with persisting hyperactivity (3.79 ± 0.02Hz, dash line in **Supplementary Figure 2D**).

On the other hand, application of anti-inflammatory drugs restored serotonin levels for mild and moderate inflammation (72.61 ± 1.28nM and 68.88 ± 1.10nM respectively, **Supplementary Figure 2E**). This was not the case in our earlier results (**Figure 3H**). Overall, anti-inflammatory drugs alone could restore serotonin levels only in those individuals where postsynaptic receptor density was reduced and when inflammation was mild or moderate. This could explain experimental studies that found anti-inflammatory drugs to be effective in depression (69, 84).

The model also predicted SCC activity — under anti-inflammatory drugs – to be 4.27 ± 0.03Hz, 4.24 ± 0.03Hz and 4.35 ± 0.03Hz for mild, moderate and severe inflammation, respectively (**Supplementary Figure 2F**). Thus, neither SSRIs nor anti-inflammatory drugs could return activity back to control levels when administered separately. As before, this normalization was seen only after combined drug administration. Then, serotonin concentration was restored to 71.53 ± 1.06nM and activity to 4.21 ± 0.03Hz for severe inflammation (**Supplementary Figures 2G,H**) (57). For mild and moderate inflammation, serotonin concentration was 80.49 ± 1.3nM and 78.27 ± 1.24nM and the activity was 4.11 ± 0.03Hz and 4.12 ± 0.03Hz respectively.

## 4 Discussion

We have presented a computational model that describes how inflammation changes extracellular serotonin levels and brain activity. Also, how brain activity, in turn, changes serotonin concentration, creating a feedback loop, leading to further changes in cortical and limbic activity, of the kind observed in depression.

Our model includes a simple, two—area cingulo-frontal circuit comprising PFC and SCC. Although these areas are not anatomically connected, they are known to be important in depression, show strong functional connectivity (43) and share a common DRN drive (85). Circuit dynamics are important in depression and its recurrence. One cause of recurrence is inflammation. This leads to changes in the HPA axis and alters cytokine levels in the cerebrospinal fluid (38, 84, 86). Cytokines are small signaling proteins that mediate communication between immune system cells and up-regulate inflammatory reactions. They are associated with multiple sclerosis and other conditions (87, 88). Cytokine inhibition reduces depressive symptoms (33). In a randomized controlled trial (33), the cytokine TNFα antagonist infliximab was administered to medically stable outpatients with major depression. Participants received infliximab or a placebo at baseline, and at weeks 2 and 6 of the 12-week trial. Those with elevated CRP levels (>5 mg/L) at baseline showed a ≥50% reduction in Hamilton Scale for Depression (HAMD) scores and a significant drop in CRP levels compared to the placebo group. Responders to infliximab also had higher baseline TNFα and soluble receptor levels than non-responders. Therefore, we focused on the cytokine TNFα that is prevalent in depression (and inflammation) and described changes under different levels of TNFα concentration.

We considered changes for three levels of inflammation: mild, moderate and severe. This taxonomy was based on the severity of inflammation-induced depression reported by (36). We formulated an index; namely, the degree of inflammation. This described inflammation severity. A higher degree of inflammation was associated with higher HAMD scores and disease severity.

(27) computed densities of 5HT2A serotonergic receptors obtained from PET scans conducted on healthy male and female participants aged 18 to 45 years. A strong correlation was found between regional PET-derived binding measures and the postmortem human brain autoradiography serotonergic receptor distribution. This enabled (27) to convert PET binding values into 5HT2A receptor densities and develop a whole brain density map of 5HT2A receptors. We defined the PFC 5HT2A serotonergic receptor density measured in (27) as $R^{PFC}$ . As SCC has an abundance of 5HT1A serotonergic receptors (45), and (27) exclusively studied 5HT2A receptors. For simplicity, we assigned the same value of $R^{PFC}$ describing the abundant 5HT2A receptors in the PFC to the 5HT1A receptors in the SCC $,R^{SCC}$ .

Our model describes changes in brain activity for mild, moderate and severe inflammation and predicted the effects of SSRIs and anti-inflammatory drugs. For high degrees of inflammation, the model predicted a reduction in cortical (PFC) resting state activity and an increase in limbic (SCC) activity, similar to recordings by (61, 63). SCC hyperactivity — of the sort predicted here — has been used to identify responders (7). In (63), an EEG study recorded the resting state peak ACC frequency of 6.5 ± 0.1Hz in depressed subjects and 6.2 ± 0.075Hz in healthy controls. An increase of about 5% in theta band activity was observed, similar to our result. Furthermore (89), noted a surge in resting rACC activity in the theta frequency band (6.5–8 Hz) as a good predictor for the degree of depression treatment response (90). performed resting state cerebral blood flow (CBF) PET scans on depressed subjects and healthy controls. They observed a decrease in CBF in the PFC region. Further, at baseline a hyperactive ACC and a hypoactive PFC was observed in Deep Brain Stimulation (DBS) responders and non-responders. However, responders demonstrated a greater magnitude reduction in prefrontal activation as compared to non-responders (90) (62). reported a reduced resting-state activity in the DLPFC and diminished activation during the induction of negative affect.

We also found that SSRI action was limited by excessive cytokine concentration observed during severe inflammation (57). Our model predicted that only for mild inflammation can SSRIs alleviate depression (91). also found lower cytokine levels in responders vs. non-responders to SSRI treatments. What distinguishes these two groups is still unclear. Our results suggest that this could be due to different levels of inflammation. Indeed, Bhattacharyya et al. (92) found increased cytokine levels in non-responders after SSRI administration.

When either antidepressants or anti-inflammatory drugs were administered in isolation, our model predicted that serotonin concentration could be restored for mild and moderate inflammation only. To restore control levels of serotonin and brain activity in severe depression, SSRIs and anti-inflammatory drugs needed to be co-administered simultaneously. This has also been observed in patients under a combined administration of anti-inflammatory drugs cyclooxygenase-2 (COX-2) inhibitors and SSRIs (93, 94). Akhondzadeh et al. (94) demonstrated the role of the anti-inflammatory agent, celecoxib, as an adjuvant with the SSRI fluoxetine. Over the six-week trial period, fluoxetine and celecoxib demonstrated superiority over fluoxetine administration alone. Similar results were obtained by Müller et al. (93) who demonstrated that celecoxib, an anti-inflammatory drug, combined with the antidepressant reboxetine showed a significant reduction in HAMD scores. Animal studies (95) also found that COX2 inhibition is associated with a reduced increase in the proinflammatory cytokines TNFα and IL1β together with lower anxiety and cognitive decline. Targeting cytokines and their signaling pathways has also been found to alleviate depression conditions (17, 96, 97).

Another inflammation effect is glutamate excitotoxicity (15, 75, 98). Activation of the kynurenine pathway activates NMDA receptors (75, 98) and contributes to excitotoxicity (15). We considered these effects here. Similar to earlier results, a combined administration of SSRIs and anti-inflammatory drugs was needed to restore control serotonin and brain activity levels in severe depression.

Our modelling follows the work of Ramirez-Mahaluf et al. (28). This work studied abnormalities in brain dynamics of depressed individuals as a result of glutamate metabolism in the ventral anterior cingulate cortex (vACC). A study by Kringelbach et al. (27) also introduced a biophysical model of serotonergic effects on brain activity. Here, we considered similar effects and their dependence on cytokine levels due to inflammation. The focus of this paper was on the disruptions caused by rise in inflammation particularly the rise in the pro-inflammatory cytokine TNFα. The variations considered were only the changes caused by variations in reuptake and synthesis in serotonin concentration and glutamate excitotoxicity due to changes in inflammation. Combining our model with Dynamic Causal Modeling will allow us to obtain patient specific parameter estimates such as neuroimaging results, similarly to (99, 100) which may be a subject for future studies and may assist with personalized treatment predictions.

It is essential to highlight that our study is exploratory in nature, presenting results that require validation in real-world settings. Our model could be extended to include other brain regions that have been implicated in depression (101), including the amygdala, hippocampus, thalamus, striatum, and the parietal lobe (102). In future work, we will include dopamine as an additional neurotransmitter system essential for understanding key depressive symptoms like cognitive impairment and lack of motivation, which has not been considered here (76, 103).

In previous work, we focused on changes in effective connectivity in depressed patients. We analyzed data from a cognitive (MSIT) task that is commonly used to assess depressive state (104). We found that individual variability was explained by the feedforward drive from sensory areas to prefrontal cortex, alongside changes in neural activity in caudal areas. Connectivity changes best on synaptic plasticity. Here, we modelled such mechanisms in detail, in terms of changes in pre and postsynaptic receptor densities. A limitation of our model is that it cannot account for the 5HT1A autoreceptor changes but only captures postsynaptic receptors associated alterations. Selectively targeting autoreceptors can increase serotonergic neurotransmission and alleviate depression symptoms (105). This acts as a brake and downregulates serotonin synthesis. This mechanism is thought to underlie delayed antidepressant response (106) — and will be pursued under our model elsewhere.

In summary, elevated TNFα levels and inflammation left unabated, can lead to recurrence of depressive episodes (76). An understanding of the intricate links between the immune system and depression could prevent this remitting, relapsing trajectory. Our model is a first step in this direction. It describes the interaction between neurotransmission underlying depression and the immune system. It can model the combined effects of antidepressants and anti-inflammatory drugs and assesses their relevance with regard to depression severity. A limitation to our model is that we have not considered long-term treatment outcomes, such as serotonin syndrome or cardiovascular risks (67, 71). This is an interesting direction that we would explore in a follow up manuscript. Further research is also needed to understand how inflammation-associated white matter damage impairs neuronal communication (107) and how TMS and DBS affects neurotransmitter systems and the immune system. We hope that our work is a step towards a mechanistic understanding of depression, the role of inflammation and potential treatments.

## Data Availability

The original contributions presented in the study are included in the article/**Supplementary Material**. Further inquiries can be directed to the corresponding authors.
